# Supplementary material for: Chitosan and Anionic Solubility Enhancer Sulfobutylether-β-Cyclodextrin-Based Nanoparticles as Dexamethasone Ophthalmic Delivery System for Anti-Inflammatory Therapy
Source: Pharmaceutics. 2024 Feb 16;16(2):277. doi: 10.3390/pharmaceutics16020277 (PMC10893235; doi:10.3390/pharmaceutics16020277)

Chitosan and anionic solubility enhancer sulfobutylether-β-cyclodextrin based nanoparticles as dexamethasone ophthalmic delivery system for anti-inflammatory therapy

Giuseppe Francesco Racaniello[a], Gennaro Balenzano[a], Ilaria Arduino[a], Rosa Maria Iacobazzi[a], Antonio Lopalco[a], Angela Assunta Lopedota[a], Hakon Hrafn Sigurdsson[b*], Nunzio Denora[a*]

[a]Department of Pharmacy – Pharmaceutical Sciences, University of Bari "Aldo Moro", 70125 Bari, Italy

[b]Faculty of Pharmaceutical Sciences, University of Iceland, Reykjavik, Iceland

* Co-Corresponding authors e-mail: nunzio.denora@uniba.it; hhs@hi.is

SUPPLEMENTARY MATERIALS

Figure S1. ^{1}H-NMR spectra related to the DEX formulation and the DEX/SBE-β-CD inclusion complex investigated at 24 hours from the start of the agitation phase (A). In the two enlargements, details of the shift experienced by the peaks related to DEX protons A and B (B) and C, D, and E (C) after the interactions with SBE-β-CD.

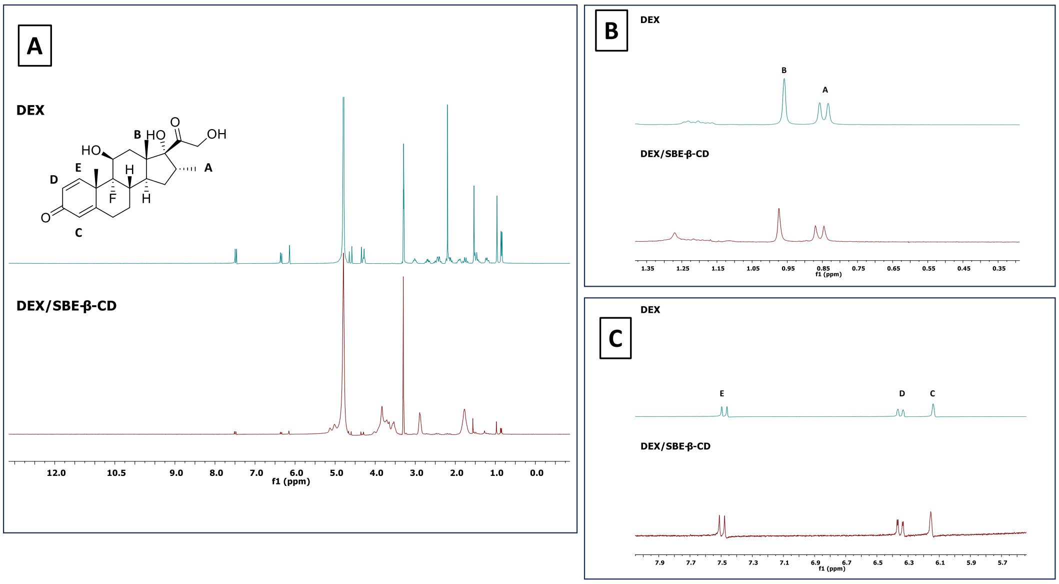

Table S1. Table indicating the shift of signals corresponding to the proton peaks of the DEX structure after interaction with SBE-β-CD.

**Proton**	δ_{DEX}	$\delta_{DEX/SBE\text{-}\beta\text{-}CD}$	$\Delta(\delta_{DEX/SBE\text{-}\beta\text{-}CD}$ **and** $\delta_{DEX})$
**A**	0.84635	0.87195	0.0256
**B**	0.9991	0.9884	−0.0107
**C**	6.1379	6.1527	0.0148
**D**	6.3472	6.36445	0.01725
**E**	7.4777	7.50745	0.02975

Figure S2. HPLC chromatogram (A) related to one of the points used to create the DEX calibration curve (B). Indication of the identified LOD and LOQ values, which represent the minimum detectable values through this calibration curve on the instrument used.

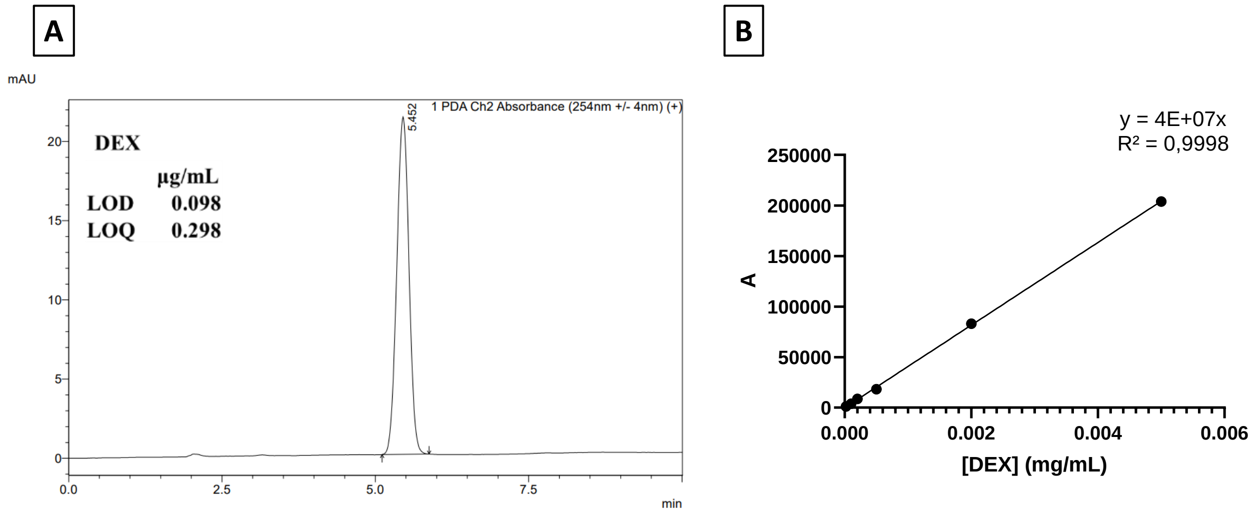

Figure S3: DSC thermoanalysis of CS and SBE-β-CD.

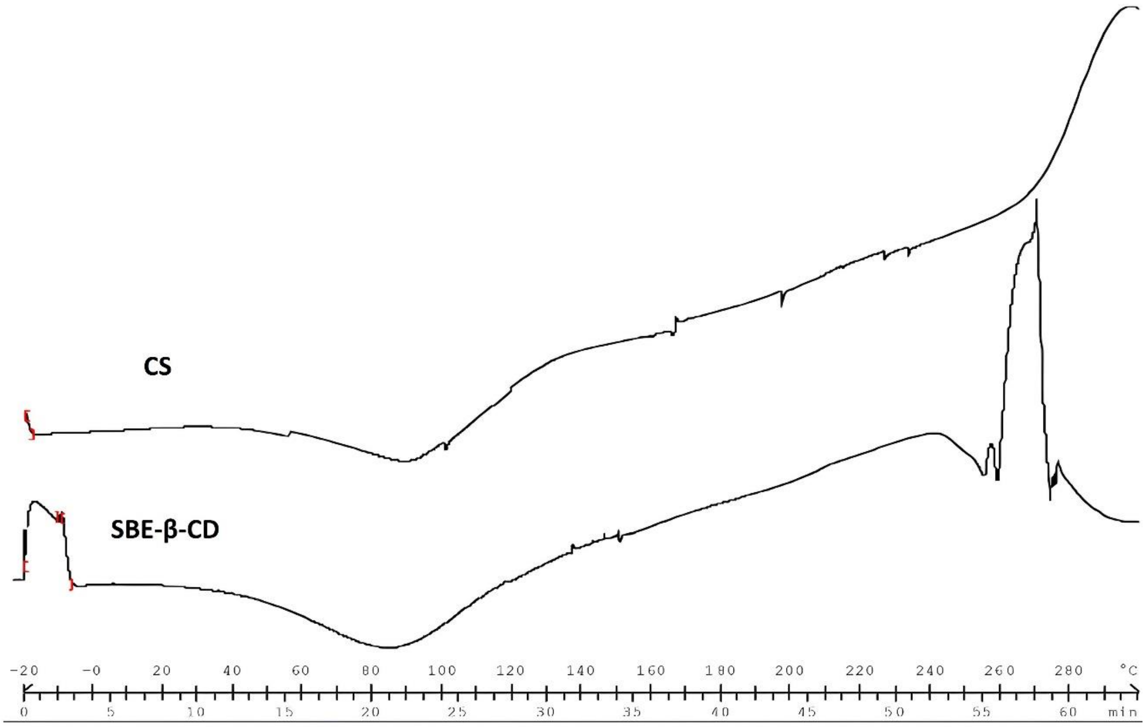

Figure S4: FT-IR spectra of CS and SBE-β-CD.

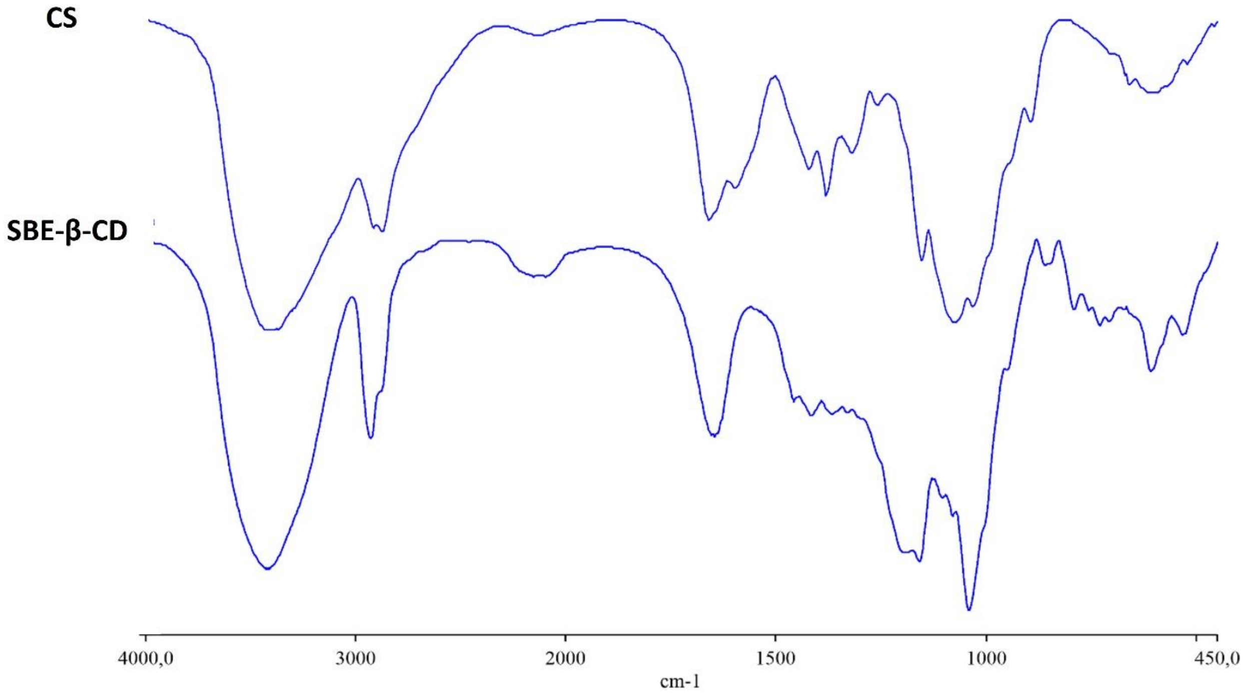

Supplement: Supplementary file 1 [file pharmaceutics-16-00277-s001.zip › pharmaceutics-2846362-supplementary.pdf]
